# Delays and Disruptions in Cancer Health Care Due to COVID-19 Pandemic: Systematic Review

**DOI:** 10.1200/GO.20.00639

**Published:** 2021-02-22

**Authors:** Rachel Riera, Ângela Maria Bagattini, Rafael Leite Pacheco, Daniela Vianna Pachito, Felipe Roitberg, Andre Ilbawi

**Affiliations:** ^1^ Centre of Health Technology Assessment, Hospital Sírio-Libanês, São Paulo, Brazil; ^2^ Discipline of Evidence-Based Medicine, Escola Paulista de Medicina (EPM), Universidade Federal de São Paulo (Unifesp), São Paulo, Brazil; ^3^ Oxford-Brazil EBM Alliance, Petrópolis, Brazil; ^4^ Centro Universitário São Camilo, São Paulo, Brazil; ^5^ Instituto do Câncer do Estado de São Paulo/HCFMUSP, São Paulo, Brazil; ^6^ Department of Noncommunicable Diseases, World Health Organization (WHO), Geneva, Switzerland; ^7^ European Society for Medical Oncology (ESMO), Lugano, Switzerland

## Abstract

**PURPOSE:**

There has been noteworthy concern about the impact of COVID-19 pandemic on health services including the management of cancer. In addition to being considered at higher risk for worse outcomes from COVID-19, people with cancer may also experience disruptions or delays in health services. This systematic review aimed to identify the delays and disruptions to cancer services globally.

**METHODS:**

This is a systematic review with a comprehensive search including specific and general databases. We considered any observational longitudinal and cross-sectional study design. The selection, data extraction, and methodological assessment were performed by two independent reviewers. The methodological quality of the studies was assessed by specific tools. The delays and disruptions identified were categorized, and their frequency was presented.

**RESULTS:**

Among the 62 studies identified, none exhibited high methodological quality. The most frequent determinants for disruptions were provider- or system-related, mainly because of the reduction in service availability. The studies identified 38 different categories of delays and disruptions with impact on treatment, diagnosis, or general health service. Delays or disruptions most investigated included reduction in routine activity of cancer services and number of cancer surgeries; delay in radiotherapy; and delay, reschedule, or cancellation of outpatient visits. Interruptions and disruptions largely affected facilities (up to 77.5%), supply chain (up to 79%), and personnel availability (up to 60%).

**CONCLUSION:**

The remarkable frequency of delays and disruptions in health care mostly related to the reduction of the COVID-19 burden unintentionally posed a major risk on cancer care worldwide. Strategies can be proposed not only to mitigate the main delays and disruptions but also to standardize their measurement and reporting. As a high number of publications continuously are being published, it is critical to harmonize the upcoming reports and constantly update this review.

## INTRODUCTION

There has been noteworthy concern about the impact of COVID-19 pandemic on essential health services including the management of cancer. In addition to being considered at higher risk for complications and worse outcomes from COVID-19,^1^ people with cancer may also experience disruptions or delays in services as a result of stressed health systems as well as increased susceptibility to the physical and psychological effects of social isolation and financial restrictions. Delays and disruptions because of the pandemic may directly or indirectly affect screening, diagnosis, treatment, palliative care, and rehabilitation of patients with cancer. Modeled and real-world data are increasingly demonstrating that an increase in cancer-related deaths will occur because of the effect of the pandemic on health systems, as recently seen in a study from the United Kingdom estimating a 20% increase in cancer-related deaths over the next 12 months.^2^

**CONTEXT**

**Key Objective**
What are the types and frequency of the delays and disruptions in cancer health care because of COVID-19 pandemic?
**Knowledge Generated**
Thirty-eight different categories of delays and disruptions with impact on treatment, diagnosis, or general health service were identified.
Delays and disruptions most investigated included reduction in routine activity of cancer services and number of cancer surgeries; delay in radiotherapy; and delay, reschedule, or cancellation of outpatient visits. Interruptions and disruptions largely affected facilities (up to 77.5%), supply chain (up to 79%), and personnel availability (up to 60%).
**Relevance**
The frequency of delays and disruptions identified was remarkable, showing a relevant impact of the pandemic on the care of patients with cancer.
Strategies can be proposed not only to mitigate the main delays and disruptions but also to standardize their measurement and reporting.

Identifying and estimating the frequency and understanding the dynamics of these limitations are critical for planning strategies to mitigate the effects of the pandemic on cancer patient outcomes. Herein, we conducted a systematic review to identify, categorize, and estimate the frequency of delays and disruptions in cancer health care as a result of the COVID-19 pandemic considering different countries and types of cancer.

## METHODS

### Study Design and Setting

This systematic review was conducted at the Centre of Health Technology Assessment, Hospital Sírio-Libanês, through a collaboration with the WHO. This study was conducted in accordance with the recommendations of Cochrane Handbook for Systematic Reviews of Interventions^3^ considering those sections relevant for a systematic review of frequency. The protocol was prospectively registered at the PROSPERO database (registration number CRD42020196708), and the reporting was written following the PRISMA statement.^4^

### Criteria for Including Studies

Taking into account the research question of interest, we considered the following study designs: observational longitudinal comparative studies (cohort or case-control), observational noncomparative studies (case series and case studies reporting the experience of a specific cancer service), cross-sectional studies (prevalence, survey, or analytical cross-sectional), controlled before-and-after studies, and uncontrolled before-and-after studies (including interrupted time series studies with two or more measures before and after the event of interest).

Reports of a single individual case were not considered. We did not consider studies assessing the effects of any intervention, strategy, or recommendation adoption for mitigating the impact of COVID-19 on cancer care since these studies are being considered in a second review from our group.

Adults or children with confirmed diagnosis or under investigation for cancer were considered as participants for this review. Any type of oncology service or hospital was considered for assessing the impact of strategies related to healthcare policies and health systems (public or private).

We considered all types of delays and disruptions in services related to cancer treatment, including delays in timely presentation, diagnosis, and referrals throughout the patient pathway, that were classified as follows: (1) delays or disruptions in diagnosis (such as delays, interruptions, and/or changes in volumes of new patients with cancer), (2) delays or disruptions in treatment (such as delays in initiating care, interruptions, and/or changes in treatment plans including reduction in services provided), and (3) general disruptions in cancer services.

Delays and disruptions reported by the primary studies should be experienced or measured by their author(s) from real-world data, instead of being estimated or obtained by a hypothetical scenario.

Structural or process factors were identified and classified as follows: (1) provider- or system-related delays or disruptions such as medicine stockouts or shortages and other health product stockouts or shortages including devices, personal protective equipment, and laboratorial or image tests; (2) reduction of personnel such as workforce shortage or reduced work time; (3) any change in cancer treatment plan and reduction in service availability including overall reduction in any activity such as medical visits, surgeries, procedures, and radiotherapy and chemotherapy sessions; (4) measures adopted by providers aiming to reduce COVID-19 risk of infection that unintentionally translated into delays or disruptions in cancer care; (5) patient-related delays or disruptions such as delays related to financial shortfall, medical comorbidities, lack of family support, and/or fear of getting infected by SARS-CoV-2; and (6) context-related delays because of social measures imposed during the pandemic, such as quarantine, lockdown, and social distancing, and COVID-19 infection.

Findings of included studies were organized as follows: (1) reported by providers, patients, centers, and/or other respondents and (2) observed and/or measured.

### Search Strategy and Selection Criteria

A comprehensive search of the literature was carried out using electronic searches with no restriction regarding date, language, or status of publication. The sensitive search strategies (Data Supplement) developed included the following databases: CINAHL (via EBSCOhost), Cochrane Library (via Wiley), EMBASE (via Elsevier), Epistemonikos, Health Systems Evidence, LILACS (via Biblioteca Virtual em Saúde), and MEDLINE (via PubMed).

Additional searches were conducted in the following COVID-19 specialized sources: McMaster Daily News COVID-19,^5^ Oxford COVID-19 Evidence Service,^6^ and WHO— Global Literature on Coronavirus Disease.^7^

Additional nonstructured searches were conducted in the following cancer specialized sources: ASCO Meeting Library (https://meetinglibrary.asco.org), ASCO Coronavirus Resources (https://www.asco.org/asco-coronavirus-information), COVID-19 and Cancer Taskforce Global Modelling Consortium,^8^ ESMO COVID-19 and Cancer,^9^ International Agency for Research on Cancer (IARC)—IARC research at the intersection of cancer and COVID-19,^10^ and Union for International Cancer Control.^11^

A search for gray literature was conducted in the OpenGrey database.^12^ Manual search was performed in the reference lists of the relevant studies.

The selection process was carried out in a two-phase process within the Rayyan platform.^13^ In the first phase, two review authors independently evaluated all titles and abstracts retrieved by the search strategies. References identified as potentially eligible were then screened at the second stage, which involved the reading of the full text to confirm its eligibility. Any divergence was solved by a third review author. Studies excluded in the second phase were presented in the excluded studies table along with the reasons for exclusions. The selection process was presented in a study flow diagram.

### Data Analysis

The data extraction process was carried out by two independent review authors, and a Microsoft Excel standard form was adopted. Divergencies in this process were reconciled by a third review author.

The methodological quality of the included studies was evaluated by two independent review authors using validated tools for each study design as follows: nonrandomized trial, quasi-randomized trial, cohort study, or case-control study—ROBINS-I^14^; controlled before-and-after study—ROBINS-I with additional issues for (controlled) before-and-after studies^14^; uncontrolled before-and-after study (including interrupted time series)—ROBINS-I with additional issues for (uncontrolled) before-after studies^14^; analytical cross-sectional study—the Joanna Briggs Institute checklist for analytical cross-sectional studies^15^ (considering the eight questions to be answered, at the discretion of the review authors, the studies were categorized as presenting high quality [scored 7 or 8], moderate quality [scored 6 or 5], or low quality [scored 4 or lower]); prevalence cross-sectional study—the Joanna Briggs Institute checklist for prevalence studies^16^; case series—NIH Quality Assessment Tool for Case Series Studies^17^; case study (service or system)—critical appraisal of qualitative studies^18^; survey—Center for Evidence-Based Management’s critical appraisal of a survey^19^ (for survey assessment, considering the 12 questions to be answered, at the discretion of the review authors, the studies were categorized as presenting high quality [scored 9-12], moderate quality [scored 5-8], or low quality [scored 4 or lower]).

Considering the context requiring a rapid answer, the authors from primary studies were not contacted for missing data.

We planned to present the results of the included studies using a narrative approach (qualitative synthesis). We created a table comprising the methodological characteristics, main findings, and funding sources from each included study. Whenever data were available, we presented the frequency of each type of delay or disruption identified by included studies and the phase of patient journey in which delay or disruption had occurred according to the outcome reported. Structural or process factors were categorized as follows: provider-related, patient-related, or context-related disruptions or delays.

For the conclusions of each delay or disruption identified, the results of the studies that had undergone a peer review editorial process were considered preferably. In the absence of such studies, no peer-reviewed reports (as those available from Web pages of research organizations) could contribute to the final conclusions of this review.

## RESULTS

### Results From Search

**FIG 1:**
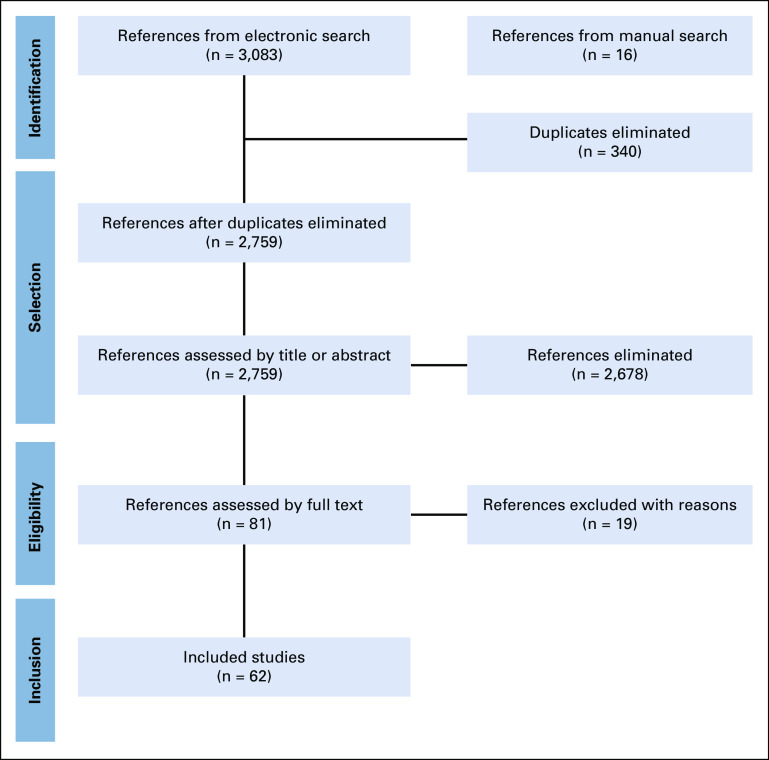
Flowchart of the process of study identification and selection.

We retrieved 3,083 references from electronic search and 16 additional references from manual search. After excluding 340 duplicates, we screened the titles and abstracts of 2,759 references, excluded 2,678 that did not comprise the eligibility criteria, and selected 81 for full text assessment. We excluded 19 studies^20-38^ with reasons (Data Supplement). Therefore, we identified 62 studies that fully met our inclusion criteria.^2,39-59,60-80,81-99^ The flowchart of the process of study identification and selection is presented in Figure 1.

### Characteristics and Results From Included Studies

**FIG 2:**
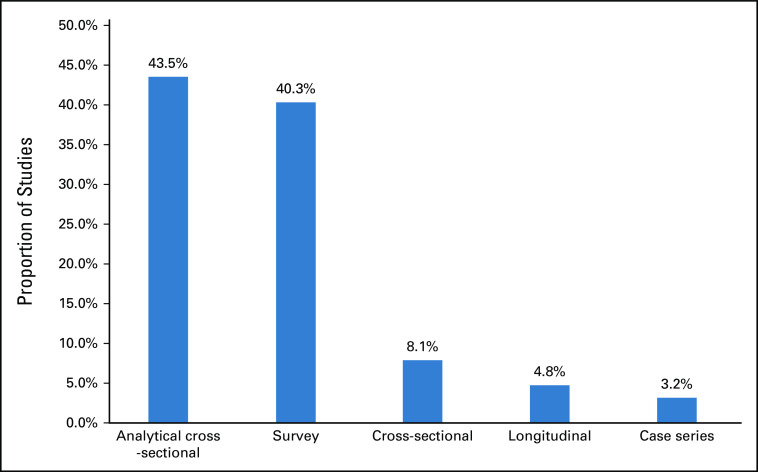
Design of included studies.

**FIG 3:**
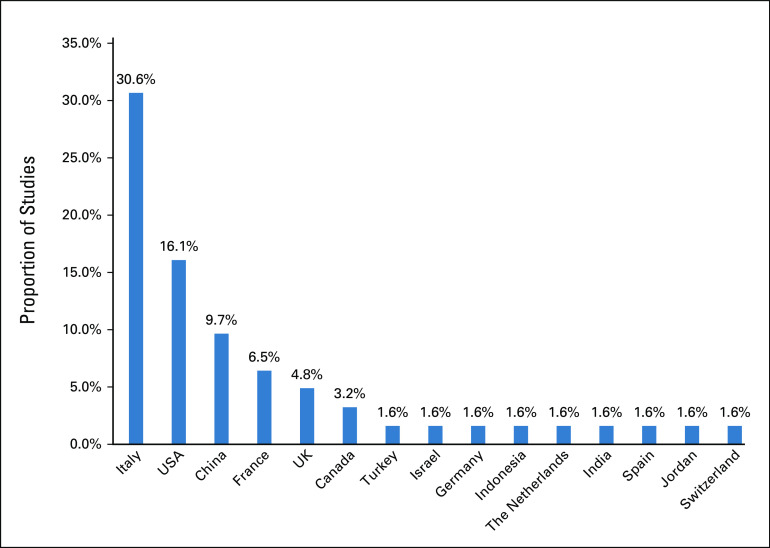
Countries considered by included studies.

**FIG 4:**
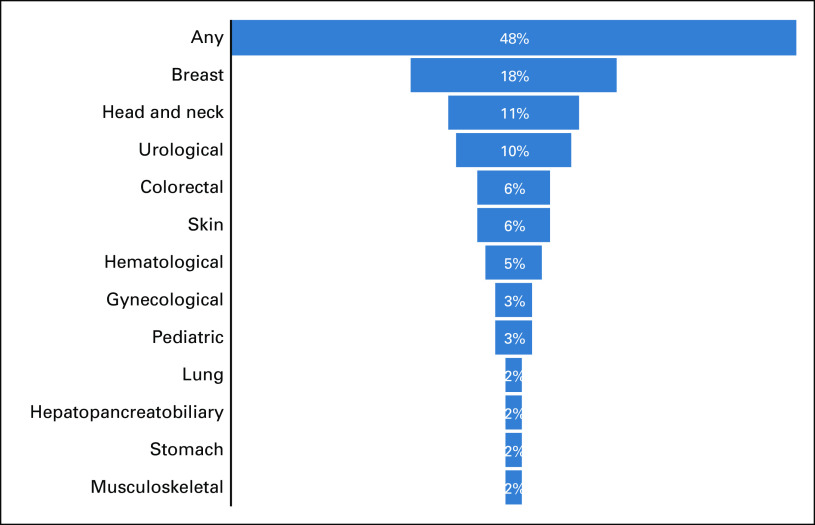
Types of cancer considered by included studies.

**FIG 5:**
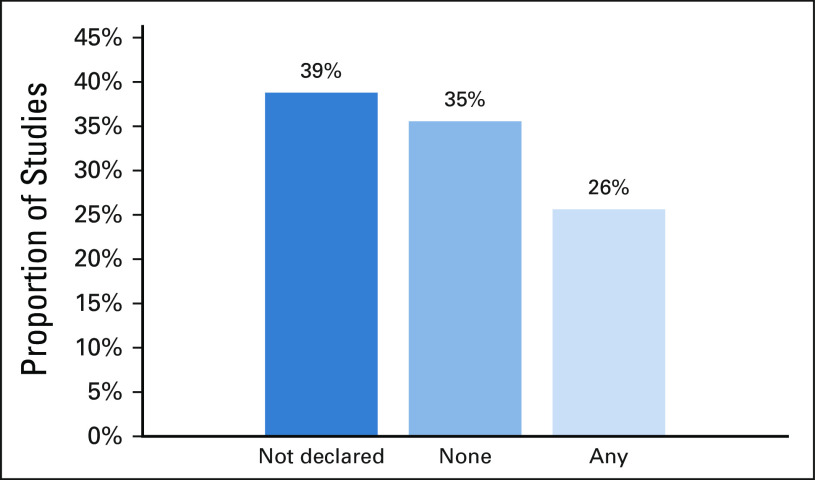
Declaration of funding source by included studies.

A summary comprising the main characteristics of the 62 included studies is presented in Figures 2-5.

Detailed information about additional characteristics and findings of these studies is presented in the Data Supplement.

### Results of Included Studies

We identified 38 main categories of delays and disruptions addressed by the included studies. Table 1 summarizes all of them and the estimated frequency. Table 2 presents the frequency of structural or process factors and the type of delay or disruption.

**TABLE 1:**
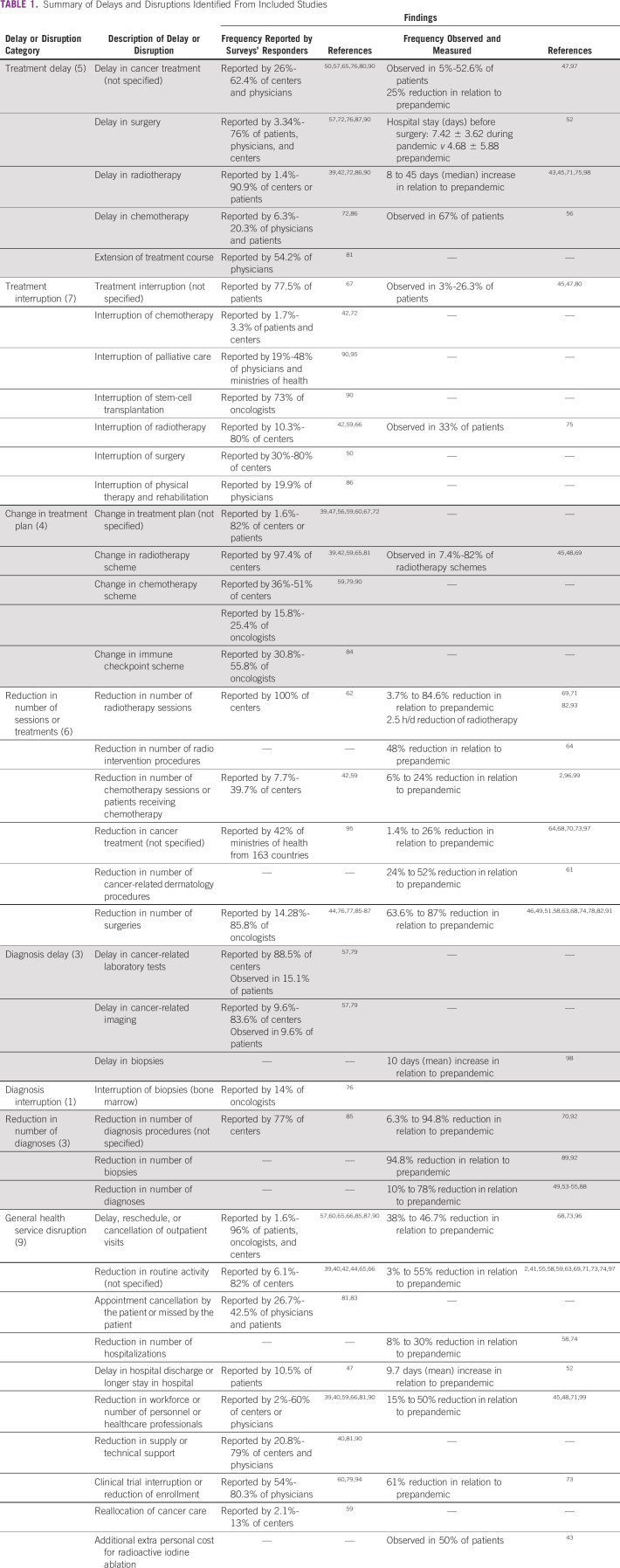
Summary of Delays and Disruptions Identified From Included Studies

**TABLE 2:**
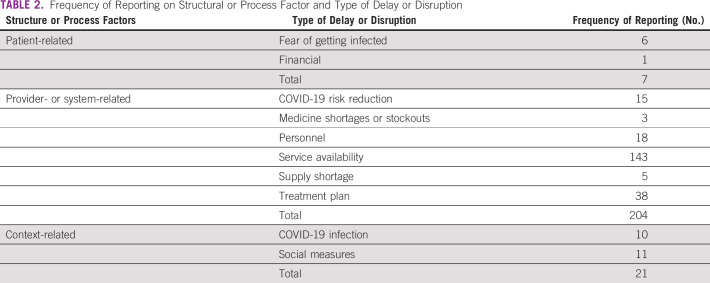
Frequency of Reporting on Structural or Process Factor and Type of Delay or Disruption

### Methodological Assessment of Studies

Methodological quality assessment of the included studies and reasons for judgment are presented in the Data Supplement. The methodological quality was considered low for case series,^43,45^ low for longitudinal studies,^47,70,80^ and moderate^2,48,75^ to low^56,93^ for cross-sectional studies.

Among analytical cross-sectional studies, the quality was considered moderate for 13^41,46,49,52,53,58,61,68,71,78,89,97,99^ and low for the remaining 14.^51,54,55,63,64,69,73,74,82,88,91,92,96,98^

For surveys, the methodological quality was considered moderate for 17^39,40,44,50,57,59,65-67,72,79,81,83,85,87,90,95^ and low for the remaining eight.^42,60,62,76,77,84,86,94^

## DISCUSSION

This systematic review included 62 studies reporting and measuring at least one delay or disruption in cancer health care because of the COVID-19 pandemic. The studies addressed 38 different categories of delays and disruptions with established or potential impact on treatment (n = 22), diagnosis (n = 7), or health-service process (n = 9).

Delays and disruptions most investigated by studies were (1) reduction in any routine activity of cancer services, including visits (n = 17 studies); (2) reduction in the number of cancer surgeries (n = 16 studies); (3) delay in radiotherapy (n = 10 studies); and (4) delay, reschedule, or cancellation of outpatient visits (n = 10 studies).

Provider- or system-related variables were the most frequently reported structural or process-related factors (n = 204), mainly because of the reduction in service availability (overall reduction in any activity such as medical visits, surgeries, procedures, radiotherapy and chemotherapy sessions, etc) (n = 143/204). This is an important finding to support further strategies aiming to mitigate the delays and disruptions in cancer care.

Interruption in any stage of treatment was reported by up to 77.5% of patients who responded to the surveys.^67^ Three longitudinal studies assessed the interruption of treatment and reported a rate of up to 26.3%.^45,47,80^ A wide variety of delays and changes in chemotherapy and radiotherapy plans were observed. The reported reasons for such delays comprised not only compliance with quarantine and/or social isolation to minimize exposure to COVID-19 but also the adoption of strategies to attend to the most urgent and highest potential volume of patients as per local or national guidelines. Many professional societies have developed guidelines for cancer patients’ treatment to support this prioritization and inform evidence-based decision making.

Except for one study that observed a 14% cancellation rate in bone marrow biopsies during the pandemic,^76^ none of the studies addressed the interruption of the diagnostic process or population-based, organized cancer screening programs. However, it is reasonable to assume that this occurred although it has not been formally evaluated or reported.

According to the studies included, the availability and maintenance of cancer services appear to be substantially affected by the pandemic. Disruption in the supply chain, which includes everything from medication to technical maintenance of imaging equipment, was reported by up to 79% of the centers interviewed.^40,81,90^ In one survey,^90^ medicine shortage was the reason for modification of chemotherapy regimens in 36% of cases, and in a second one,^40^ 43% of centers reported participants’ difficulty or reduced access to anticancer medication.

Personnel with reduced members (in some cases up to 50%) were reported by up to 60% of respondents in the studies. Quarantine and displacement of the workforce to COVID-19 care were responsible for this reduction.^39,40,45,59,90^ None of the studies assessed the impact of staff shortages on the workload of those who continued to work in cancer care.

In the face of such scarcity, the cancer-related hospitalization rate was also reduced by up to 30% with respect to the prepandemic period.^58,74^

Clinical trials in oncology, which historically represent a remarkable possibility of offering to many patients a therapeutic option, have also been discontinued or stopped enrolling participants according to more than 80% of the data obtained by the studies in this review. There was a 65% reduction in clinical trial activities in relation to the prepandemic period.

All the findings presented above come mainly from surveys, analytical cross-sectional studies, or were estimated by cross-sectional studies and case series. None of them were at high methodological quality using the specific tools and criteria.

Most of the studies used a similar period before the pandemic as comparators. The absence of a concomitant control group increases the risk of bias, but it is recognized that a reliable concurrent group not exposed to the virus and pandemic is not feasible. Nonetheless, this fact does not reduce the risk of bias, and the interpretation and generalization of results should consider temporal distortions, particularly as countries transition through the different phases of the pandemic that may influence the estimates for the 27 analytical cross-sectional studies included.

We highlight the pronounced clinical and methodological heterogeneity among the included studies. The studies were conducted in several countries with various public and private health systems and policies. Twelve different cancer conditions were considered, including early-stage and/or metastatic disease, and the data were collected during distinct pandemic stages. Therefore, most of the disruptions and delays identified in this review probably do not occur homogeneously between or perhaps within different countries, scenarios, and/or types of cancers. This heterogeneity may have contributed to the wide range in the frequencies reported by the studies. An additional concern for the decision-making process is that most of the measures adopted to control the pandemic and reduce the risk of SARS-CoV-2 infection could have been unintentionally associated with disadvantages to cancer care by reducing or limiting both the offer and access to health services.

Substantial heterogeneity is also noted in the reported outcome measures. In this review, 38 categories of delays and disruptions were measured, reinforcing the need to establish prioritized data sets for reporting and monitoring progress over time. The recent WHO Maintaining essential health services: operational guidance for the COVID-19 context provides a list of sample indicators for monitoring the maintenance of essential health services during the COVID-19 pandemic, which includes new cancer diagnoses.^100^

With respect to the generalizability of these findings, we highlight that the majority of studies are from high-income countries (HICs) and low- and middle-income countries (LMICs) are underrepresented in the included studies (16.1% of studies). It is expected that the frequency of delays and disruptions in cancer care due to the COVID-19 pandemic may be higher in LMICs given baseline capacity to deliver essential health services in cancer and absorptive capacity.

This systematic review has some limitations. Many of the COVID-19 studies are being published through a fast-track process as preprint and/or without a peer review rather than being available in indexed databases. This fact increases the probability of missing studies that would potentially be included. We minimized this risk by searching on several databases and complementing the search with a broad nonstructured search in relevant data sources.

To the best of our knowledge, this is the first complete systematic review that attempted to identify the frequency of delays and disruptions in cancer health care. We anticipate our findings would improve the understanding of current cancer care scenarios and support the proposition and assessment of specific strategies to mitigate identified delays and disruptions.

As key messages from our findings, we highlight the following: (1) studies to date have shown diverse and substantial impact on cancer services, (2) studies tied to address the primary outcomes of interest, such as survival and morbidity, represent a gap in the literature and an unmet need, (3) many studies are highlighting the adaptability of treatment services or plans (particularly in HICs), however do not report context factors such as geographic accessibility as a result of social distance measures, (4) scientific community must consider long-term impacts of current disruptions through longitudinal studies, and (5) harmonized data sets and reporting trends over time are required.

In conclusion, this review identified the main delays and disruptions in cancer health care because of COVID-19 addressed and measured by 62 primary studies. Overall, 38 categories of delays and disruptions were identified as outcomes of interest. Structural or process factors related to provider were the most frequently reported by studies, mainly because of the reduction in service availability.

The frequency identified by the studies was remarkable, showing a relevant impact of the pandemic on the care of patients with cancer. From now and on the basis of these findings, strategies can be proposed to mitigate the main delays and disruptions. Nonetheless, it is indispensable to be aware that, under the pandemic context, a high number of publications continuously made available in a short period of time reinforces the importance of constantly updating this systematic review.
